# Engineered *Bacillus subtilis* for the de novo production of 2′-fucosyllactose

**DOI:** 10.1186/s12934-022-01838-w

**Published:** 2022-06-02

**Authors:** Quanwei Zhang, Zhenmin Liu, Hongzhi Xia, Ziyang Huang, Yonglian Zhu, Linfeng Xu, Yanfeng Liu, Jianghua Li, Guocheng Du, Xueqin Lv, Long Liu

**Affiliations:** 1 Key Laboratory of Carbohydrate Chemistry and Biotechnology, Ministry of Education, Jiangnan University, Wuxi, 214122 China; 2 Science Center for Future Foods, Jiangnan University, Wuxi, 214122 China; 3 State Key Laboratory of Dairy Biotechnology, Shanghai Engineering Research Center of Dairy Biotechnology, Dairy Research Institute, Bright Dairy & Food Co., Ltd, Shanghai, 200436 China; 4 Nantong Licheng Biological Engineering Co., Ltd, Shanghai, 200000 China; 5 Yixing Institute of Food Biotechnology Co., Ltd, Yixing, 214200 China

**Keywords:** *Bacillus subtilis*, De novo pathway, 2′-fucosyllactose, GDP-_*L*_-fucose

## Abstract

**Background:**

The most abundant human milk oligosaccharide in breast milk, 2′-fucosyllactose (2′-FL), has been approved as an additive to infant formula due to its multifarious nutraceutical and pharmaceutical functions in promoting neonate health. However, the low efficiency of de novo synthesis limits the cost-efficient bioproduction of 2′-FL.

**Results:**

This study achieved 2′-FL de novo synthesis in a generally recognized as safe (GRAS) strain *Bacillus subtilis*. First, a de novo biosynthetic pathway for 2′-FL was introduced by expressing the *manB*, *manC*, *gmd*, *wcaG,* and *futC* genes from *Escherichia coli* and *Helicobacter pylori* in *B. subtilis*, resulting in 2′-FL production of 1.12 g/L. Subsequently, a 2′-FL titer of 2.57 g/L was obtained by reducing the competitive lactose consumption, increasing the regeneration of the cofactor guanosine-5′-triphosphate (GTP), and enhancing the supply of the precursor mannose-6-phosphate (M6P). By replacing the native promoter of endogenous *manA* gene (encoding M6P isomerase) with a constitutive promoter P7, the 2′-FL titer in shake flask reached 18.27 g/L. The finally engineered strain BS21 could produce 88.3 g/L 2′-FL with a yield of 0.61 g/g lactose in a 3-L bioreactor, without the addition of antibiotics and chemical inducers.

**Conclusions:**

The efficient de novo synthesis of 2′-FL can be achieved by the engineered *B. subtilis*, paving the way for the large-scale bioproduction of 2′-FL titer in the future.

**Supplementary Information:**

The online version contains supplementary material available at 10.1186/s12934-022-01838-w.

## Introduction

Studies on the intestinal microbiome in recent years have found that the composition of intestinal microorganisms impacted on human health [1–3]. Breast milk is an important food for infants; its special ingredients, such as oligosaccharides, antibodies, and vitamins, greatly influence the composition of the infant’s gut microbiome, thus affecting the infant’s health. Human milk oligosaccharides (HMOs) are the third most abundant solid substance in breast milk, following lactose and fat [4]. 2′-Fucosyllactose (2′-FL) is the most abundant HMOs, accounting for ~ 30% of the total HMOs [5, 6], and is composed of L-fucose, D-galactose, and D-glucose units. 2′-FL has gained much attention in recent years given its bioactive nature [5], which offers important health and economic benefits. Although indigestible to human infants [7], it plays a crucial role in developing the immune system, regulating the intestinal flora, and suppressing pathogenic infections [8–12]. These known bioactivities and their potential value make HMOs, especially 2′-FL, an attractive research target for preventing or treating diseases in infants and adults. To date, 2′-FL has been approved as a prebiotic for use in infant formula by the U.S. Food and Drug Administration, the European Food Safety Authority, and the Australian Therapeutic Goods Administration.

**Fig. 1:**
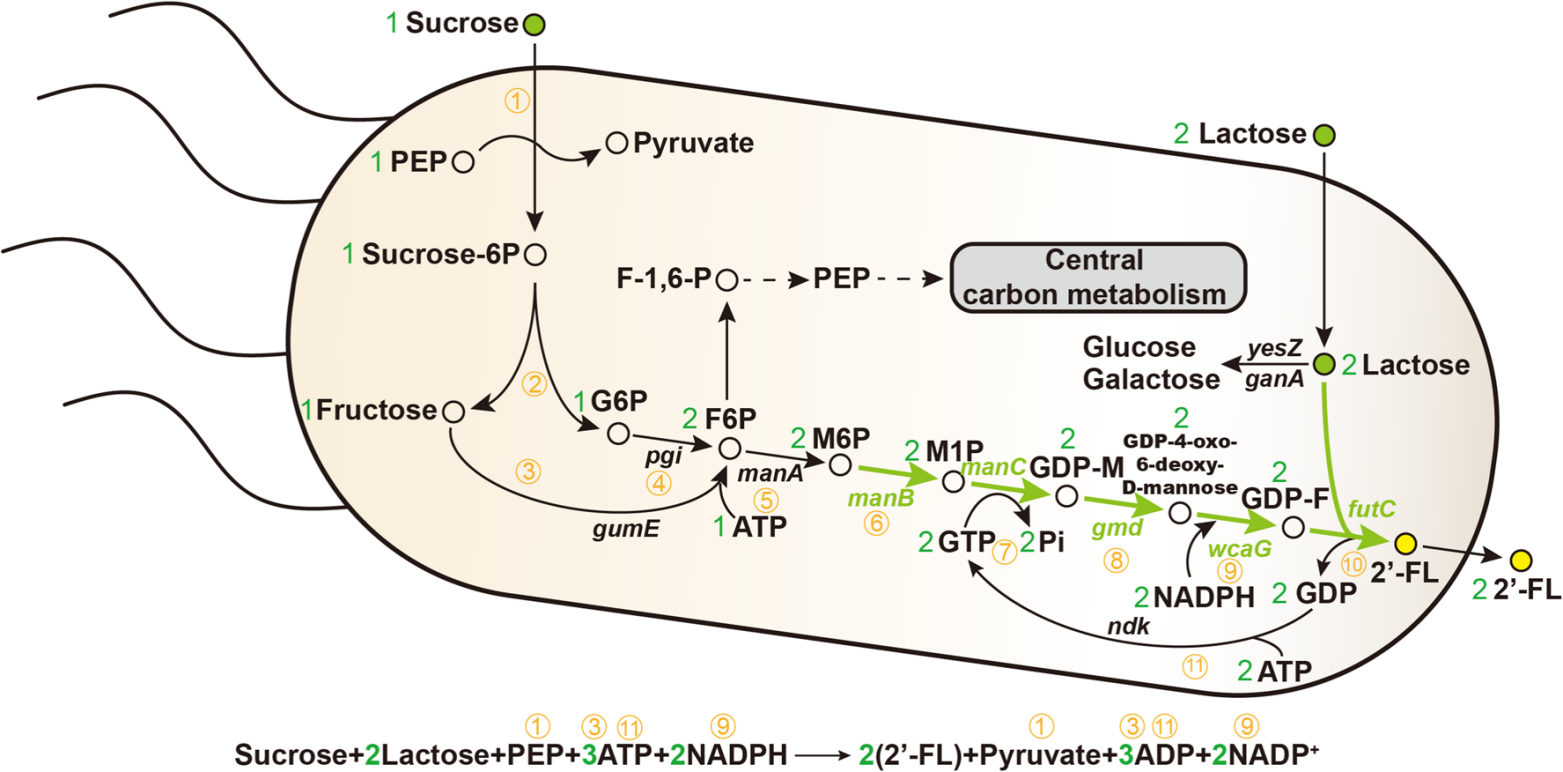
Engineered *B. subtilis* strain for de novo synthesis of 2ʹ-FL using sucrose and lactose as carbon sources. Green arrows and genes indicate a heterogenous 2′-FL de novo biosynthetic pathway. Serial numbers represent the corresponding metabolic reactions. Abbreviations: F-1,6-P, fructose-1, 6-diphosphate; F6P, fructose-6-phosphate; G6P, glucose-6-phosphate; GDP-F, GDP-_*L*_-fucose; GDP-M, GDP-mannose; M1P, mannose-1-phosphate; PEP, phosphoenolpyruvate; sucrose-6P, sucrose-6-phosphate

Currently, two promising routes are available to produce 2′-FL: chemical synthesis and biosynthesis [13, 14]. The chemical synthesis of 2′-FL is accomplished through multistep protection and deprotection reactions. This method is inefficient and uses toxic chemicals, rendering the chemical method unfit for common use [14]. The reaction steps for 2′-FL biosynthesis are simpler and more environmentally friendly than its chemical synthesis. The two known 2′-FL biosynthetic pathways are the salvage and de novo synthesis pathway. Both pathways are involved in lactose fucosylation (α-1,2-fucosyltransferase; FutC), the difference being in the synthesis pathway of GDP-_*L*_-fucose. The salvage pathway was first discovered in *Bacteroides fragilis*. It catalyzes GDP-_*L*_-fucose production from the substrate fucose by the fucokinase/GDP-_*L*_-fucose phosphorylase [15]. The de novo pathway is part of mannose metabolism, which starts from mannose-6-phosphate (M6P) and is catalyzed by four enzymes, namely, phosphomannomutase, mannose-1-phosphate guanylyltransferase, GDP-mannose-4, 6-dehydratase, and GDP-_*L*_-fucose synthase, to generate GDP-_*L*_-fucose (Fig. 1). The de novo pathway utilizes a low-cost carbon source, such as glucose and sucrose, instead of fucose; it is more economical and industrially applicable. *Escherichia coli*, *Saccharomyces cerevisiae*, and *Corynebacterium glutamicum* have been engineered to produce 2′-FL using the de novo pathway [16–18]. Besides the abovementioned microorganisms, *Bacillus subtilis* has also been selected as a host for producing functional nutraceuticals due to its generally recognized as safe (GRAS) status [19]. In previous studies, we have achieved the biosynthesis of 2′-FL in the engineered *B. subtilis* by using the salvage pathway [20]. However, there are few reports about the de novo biosynthesis of 2′-FL in the engineered *B. subtilis* strains.

In this study, we achieved 2′-FL de novo synthesis by introducing the de novo pathway of GDP-fucose and FutC in *B. subtilis*. First, *B. subtilis* was tested for 2′-FL tolerance to determine whether this host has the potential to produce high concentrations of 2′-FL. Then, the heterologous 2′-FL de novo synthesis pathway derived from *E. coli* and *Helicobacter pylori* was introduced in *B. subtilis*, and a 2′-FL titer of 1.12 g/L was obtained. Next, the supply of the precursors lactose and M6P and the cofactor (guanosine-5′-triphosphate, GTP) was increased, and the titer was increased by 129%. By optimizing the promoter of M6P isomerase, the 2′-FL titer of 18.27 g/L was obtained in the shake flask using sucrose and lactose as carbon sources. Finally, in a 3-L bioreactor, the 2′-FL titer of the finally engineered strain BS21 reached 88.3 g/L, the highest 2′-FL titer reported so far.

## Methods

### Plasmids, strains, and culture conditions

The strains used in this study are listed in Table 1. All plasmid constructions were performed using *E. coli* DH5α. The engineered BS strain (*B. subtilis*-P_*xylA*_-*comK*::*comK*) was used as a host strain for the heterologous synthesis of 2′-FL. Luria–Bertani (LB) medium (10 g/L tryptone, 5 g/L yeast extract and 10 g/L NaCl) was used to culture *E. coli* DH5α and the engineered *B. subtilis*. The minimal medium with the following composition was used: 17.1 g/L Na_2_HPO_4_·12H_2_O, 3 g/L KH_2_PO_4_, 1 g/L NH_4_Cl, 0.5 g/L NaCl, 0.0001 M CaCl_2_, 0.001 M MgSO_4_, 0.005 mM FeCl_3_, 1 mg/L MnSO_4_·4H_2_O, 1.7 mg/L ZnCl_2_, 0.43 mg/L CuCl_2_·2H_2_O, 0.6 mg/L CoCl_2_·6H_2_O, 0.6 mg/L Na_2_MoO_4_·2H_2_O. When needed, antibiotics were added as follows: kanamycin 50 μg/mL, chloromycetin 5 μg/mL, ampicillin 100 μg/mL. To cultivate *B. subtilis* or *E. coli* strains, a single colony was inoculated in a shake tube with LB medium, as described earlier, at 37 °C and 220 rpm for 8–10 h. A Competent Cell Preparation Kit (Takara Biomedical Technology, Beijing, China) was used to prepare *E. coli* DH5α competent cells. A previously reported method was followed for the preparation of *B. subtilis* competent cells [20]. To examine the tolerance of *B. subtilis* to 2′-FL, *B. subtilis* was cultured in 200 μL of LB media containing different concentrations of 2′-FL (0 g/L, 10 g/L, 20 g/L and 30 g/L) in 96-well plates (Corning 3603) at 37 °C and 750 rpm. The optical density (OD_600_) was determined at 12-h intervals.

**Table 1:** Strains used in this study

Strains	Characterization	Source
*B. subtilis* 168	Standard strain, starting strain	lab stock
*E. coli* DH5α	Cloning host	lab stock
*E. coli* BL21 (DE3)	Standard strain	lab stock
BS	*B. subtilis* 168, P_*xylA*_-*comK*::*comK*	lab stock
BS0	BS, P_43_-*manB*, P_43_-*manC*, P_43_-*gmd*, P_43_-*wcaG*, P_43_-*futC*	this work
BS-MB	BS0-pP43NMK-*manB*-His	this work
BS-MC	BS0-pP43NMK-*manC*-His	this work
BS-GD	BS0-pP43NMK-*gmd*-His	this work
BS-WG	BS0-pP43NMK-*wcaG*-His	this work
BS1	BS0, P_43_-*manB*, P_43_-*gmd*, P_43_-*wcaG*, P_43_-*futC*	this work
BS2	BS1, P_*xylA*_-*manC*	this work
BS3	BS2, Δ*yesZ*	this work
BS4	BS2, Δ*ganA*	this work
BS5	BS2, Δ*yesZ*, Δ*ganA*	this work
BS6	BS5, P_native_-*ndk*	this work
BS7	BS5, P_*xylA*_-*ndk*	this work
BS8	BS5, P_*hbs*_-*ndk*	this work
BS9	BS5, P_43_-*ndk*	this work
BS10	BS8, P_native_-*yvyI*	this work
BS11	BS8, P_43_-*yvyI*	this work
BS12	BS8, P_native_-*manA*	this work
BS13	BS8, P_43_-*manA*	this work
BS14	BS12, P_1_-*manC*:: P_*xylA*_-*manC*	this work
BS15	BS12, P_2_-*manC*:: P_*xylA*_-*manC*	this work
BS16	BS12, P_3_-*manC*:: P_*xylA*_-*manC*	this work
BS17	BS12, P_4_-*manC*:: P_*xylA*_-*manC*	this work
BS18	BS12, P_5_-*manC*:: P_*xylA*_-*manC*	this work
BS19	BS12, P_6_-*manC*:: P_*xylA*_-*manC*	this work
BS20	BS12, P_7_-*manC*:: P_*xylA*_-*manC*	this work
BS21	BS20, P7-*manA*::P_native_-*manA*	this work

### Plasmid construction and DNA manipulation

All plasmids are listed in Additional file 1: Table S1, and the primers and promoters are listed in Additional file 1: Table S2. A CRISPR/Cpf1 genome editing approach was used to perform gene knockout or overexpression in *B. subtilis*, as reported previously [21]. First, the pcrF19NM plasmid was digested using *Bsa*I. T4 DNA ligase was added to connect the crRNA fragment with linearized pcrF19NM plasmid to construct a gene-editing plasmid. In addition, the plasmid with gene-editing DNA fragments was constructed using the Seamless Cloning Kit (Beyotime Biotechnology, Shanghai, China) according to the manufacturer’s instructions. The plasmids of pHT-XCR6 and pcrF19NM-XXX (XXX is the name of the corresponding gene, such as *manB*, *manC*, *gmd*, *wcaG*, and *futC*) were sequentially transformed into *B. subtilis*, and DNA modification was performed. The validated positive colony was inoculated into the LB medium containing 0.005% sodium dodecyl sulfate (SDS) for plasmid curing. Finally, plasmid-free strains were obtained through antibiotic screening. The specific genome integration sites are as follows: *manB* gene was integrated between the *yqiG* and *spo0A* genes, and the *manc* gene was integrated between the *yxkC* and *galE*; the *gmd* gene was integrated between the *ybcI* and *ybzH*; the *wcaG* gene was integrated between the *ybbU* and *alkA*; and the *futC* gene was integrated between the *rpsD* and *tyrS* genes.

### Analytical methods

The 2′-FL and xylose concentrations were analyzed using high-performance liquid chromatography (HPLC) system (Agilent Technologies 1260 Series) equipped with a Rezex ROA Organic Acid H + (8%) Column (Phenomenex, Torrance, CA, USA). The column and the refractive index detector temperature were set at 50 °C, and the mobile phase was 10 mM H_2_SO_4_ at a flow rate of 0.5 mL/min at 40 °C. The sucrose and lactose concentrations were analyzed using the HPLC system (Agilent Technologies 1260 Series) equipped with an XBridge BEH Amide Column (Waters, Milford, MA, USA). The column and the refractive index detector temperature were set at 35 °C, and the mobile phase was 75% acetonitrile at a flow rate of 1.0 mL/min at 35 °C. The liquid chromatography/mass spectrometry (LC/MS) matrix-assisted laser desorption/ionization time-of-flight system (Waters) was used to identify 2′-FL according to a previous study [22], and the mass range was 100–700 m*/z*. The relative transcription levels of the regulated genes were determined by quantitative real-time polymerase chain reaction (qRT-PCR) as described previously [23], and the *rpsJ* gene was used as the internal standard [24]. All experiments were independently carried out at least thrice.

### Batch fermentation in shake flasks

During shake flask fermentation, the fermentation medium with the following composition was used: 6 g/L tryptone, 12 g/L yeast extract, 12.5 g/L K_2_HPO_4_·3H_2_O, 2.5 g/L KH_2_PO_4_, and 10 mL/L trace metal solution (composition: 4 g/L FeSO_4_·7H_2_O, 4 g/L CaCl_2_, 1 g/L MnSO_4_·H_2_O, 0.2 g/L NaMoO_4_·2H_2_O, 0.2 g/L ZnSO_4_·7H_2_O, 0.1 g/L AlCl_3_·6H_2_O, 0.1 g/L CuCl_2_·2H_2_O, and 0.05 g/L H_3_BO_4_). Sucrose and lactose were sterilized separately and added to the sterilized shake flask to a final concentration of 20 and 10 g/L (changed to 80 and 20 g/L after optimization), respectively. If the induction of the xylose promoter P_*xylA*_ was required, 20 g/L xylose was added as an inducer. *B. subtilis* strains were cultivated on LB solid culture medium for 10–12 h at 37 °C, and a single colony was inoculated into 20 mL liquid LB medium in 250 mL shake flasks at 37 °C and 220 rpm for 10–12 h. The seed cultures were further inoculated into 30 mL of the fermentation medium at the rate of 10% in a 250 mL baffled flask at 37 °C and 220 rpm for 72 h, and three replicates were set for each strain. Sampling was performed every 12 h for OD_600_, 2′-FL, carbon source, and by-products measurements.

### Fed-batch culture in 3-L bioreactor

The fermentation medium used for the fed-batch culture consisted of 24 g/L yeast extract, 24 g/L tryptone, 13.1 g/L K_2_HPO_4_·3H_2_O, 3 g/L KH_2_PO_4_, 6.7 g/L urea and 10 mL/L trace metal solution (composition: 4 g/L FeSO_4_·7H_2_O, 4 g/L CaCl_2_, 1 g/L MnSO_4_·H_2_O, 0.2 g/L NaMoO_4_·2H_2_O, 0.2 g/L ZnSO_4_·7H_2_O, 0.1 g/L AlCl_3_·6H_2_O, 0.1 g/L CuCl_2_·2H_2_O, and 0.05 g/L H_3_BO_4_). Seed culture was carried out in baffled 500 mL shake flasks containing 75 mL LB medium at 37 °C with shaking at 220 rpm for 12 h. The seed culture (75 mL) was inoculated into a 3-L bioreactor (T&J Bioengineering Co., Ltd., Shanghai, China) with an initial 1.5 L fermentation medium. The pH was kept at 6.0–6.5 by adding 29% NH_3_·H_2_O, and the temperature was maintained at 37 °C. The aeration rate and the agitation speed were 2.0 vvm and 1000 rpm, respectively. Sucrose and lactose were maintained at 10–30 g/L and 10–20 g/L by feeding concentrated sucrose (800 g/L) and lactose (300 g/L), respectively.

### Statistical analysis

All data were the average of three independent studies with standard deviations. The * and ** indicate P < 0.05 and P < 0.01 relative to control, respectively.

## Results and discussion

### 2′-FL tolerance of the ***B. subtilis*** strain

**Fig. 2:**
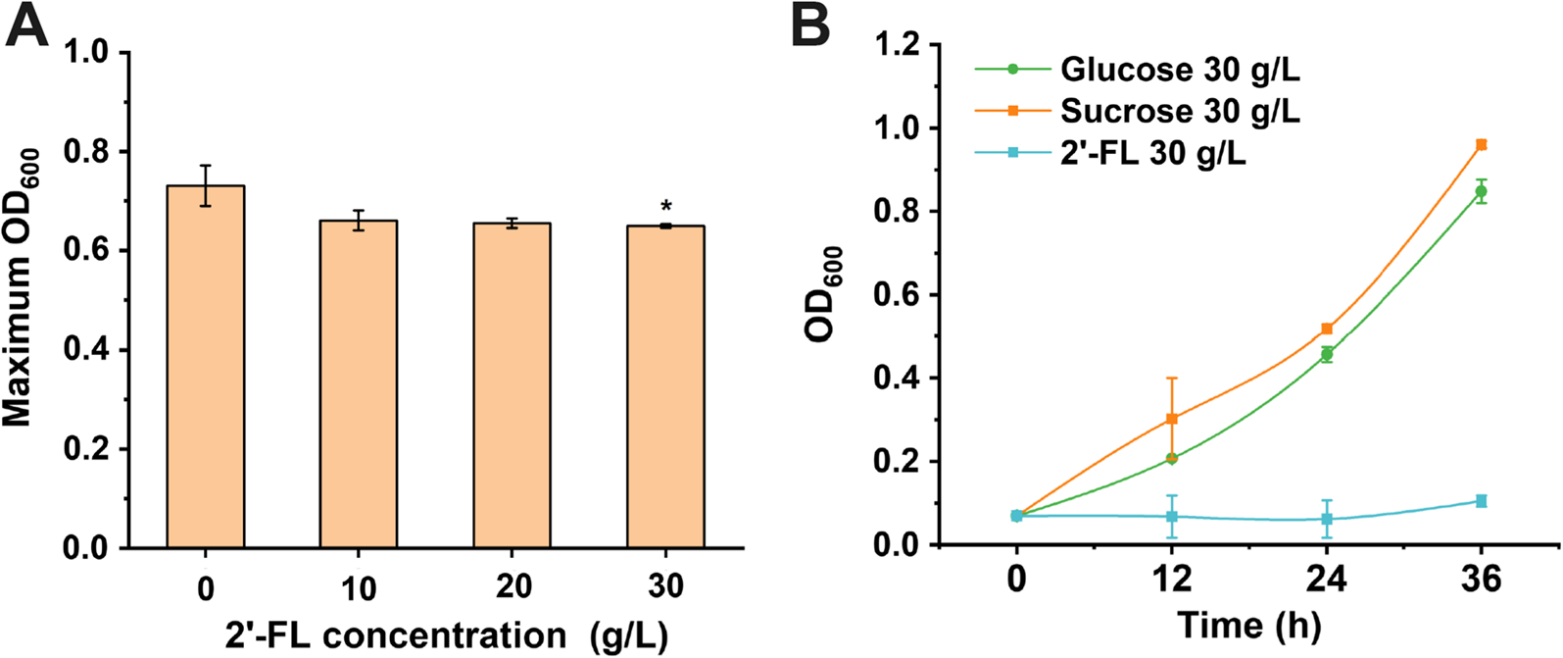
2′-FL tolerance of *B. subtilis*. **A** Maximum OD600 of *B. subtilis* in different 2′-FL concentrations (96-well plate). All data were the average of three independent studies with standard deviations. *P < 0.05; *P < 0.01. **B** Cell growth of *B. subtilis* in minimal medium with 2′-FL as the sole carbon source. Glucose and sucrose were used as controls, respectively

Previous studies indicated that 2′-FL had growth-inhibiting and adhesion-reducing effects on certain microorganisms [25, 26], and thereby the tolerance of *B. subtilis* to 2′-FL needs to be examined. *B. subtilis* was cultured in LB medium containing different concentrations of 2′-FL with 96-well plates. The maximum OD_600_ of all experimental groups added with 10, 20, and 30 g/L 2′-FL decreased by 9.62%, 10.40%, and 11.17%, respectively (Fig. 2A). Although the maximum OD_600_ was slightly lower than that in the control group without 2′-FL addition, it did not significantly decrease with higher 2′-FL concentrations. In order to identify whether the tolerance of *B. subtilis* to 2′-FL comes from its ability to utilize 2′-FL, *B. subtilis* was cultured in minimal medium with 2′-FL as the sole carbon source, and the minimal medium with glucose or sucrose as the sole carbon source was used as the control. As shown in Fig. 2B, *B. subtilis* cannot grow in the minimum medium with 2′-FL as the sole carbon source, while can grow normally in the presence of glucose or sucrose. These results suggested that *B. subtilis* could be used as a host for 2′-FL production.

### 2′-FL production by introducing the de novo pathway

GDP-_*L*_-fucose is a key precursor for 2′-FL biosynthesis [27]. The de novo pathway could utilize low-cost carbon sources (glucose, sucrose, and so on) to synthesize 2′-FL. To produce 2′-FL utilizing sucrose and lactose, the de novo pathway was constructed by introducing *manB*, *manC*, *gmd*, and *wcaG* genes from *E. coli* BL21 (DE3) for synthesizing GDP-_*L*_-fucose and the *futC* gene from *H. pylori* into *B. subtilis* 168 (Fig. 1). To express the heterologous genes efficiently in *B. subtilis*, the original promoters of *manB*, *manC*, *gmd*, *wcaG*, and *futC* genes were respectively replaced by constitutive promoter P_43_ and integrated into the genome of *B. subtilis*, yielding the strain BS0. The shake flask culture of BS0 was performed, while the extracellular 2′-FL and intracellular GDP-_*L*_-fucose cannot be detected, implying that one or more genes in the GDP-_*L*_-fucose synthesis pathway were not normally transcribed or translated. In addition, protein structure instability may also lead to the above results.

To explore the reasons for the above unexpected results, the plasmid pP43NMK was used to respectively express the *manB*, *manC*, *gmd*, and *wcaG* genes. Four strains (BS-MB, BS-MC, BS-GD, and BS-WG) were constructed to verify the expressions of phosphomannomutase (ManB; 50.19 kDa), mannose-1-phosphate guanylyltransferase (ManC; 53.81 kDa), GDP-mannose-4,6-dehydratase (Gmd; 42.05 kDa), and GDP-_*L*_-fucose synthase (WcaG; 36.15 kDa). SDS–polyacrylamide gel electrophoresis results showed that BS-MB, BS-GD, and BS-WG strains displayed single bands of approximate 51, 42, and 37 kDa, respectively (Additional file 1: Fig. S1), while the gel electrophoresis result of strain BS-MC was similar to the control group. The aforementioned results suggested that the target genes in strains BS-MB, BS-GD, and BS-WG were transcribed and translated but not the target gene in strain BS-MC.

**Fig. 3:**
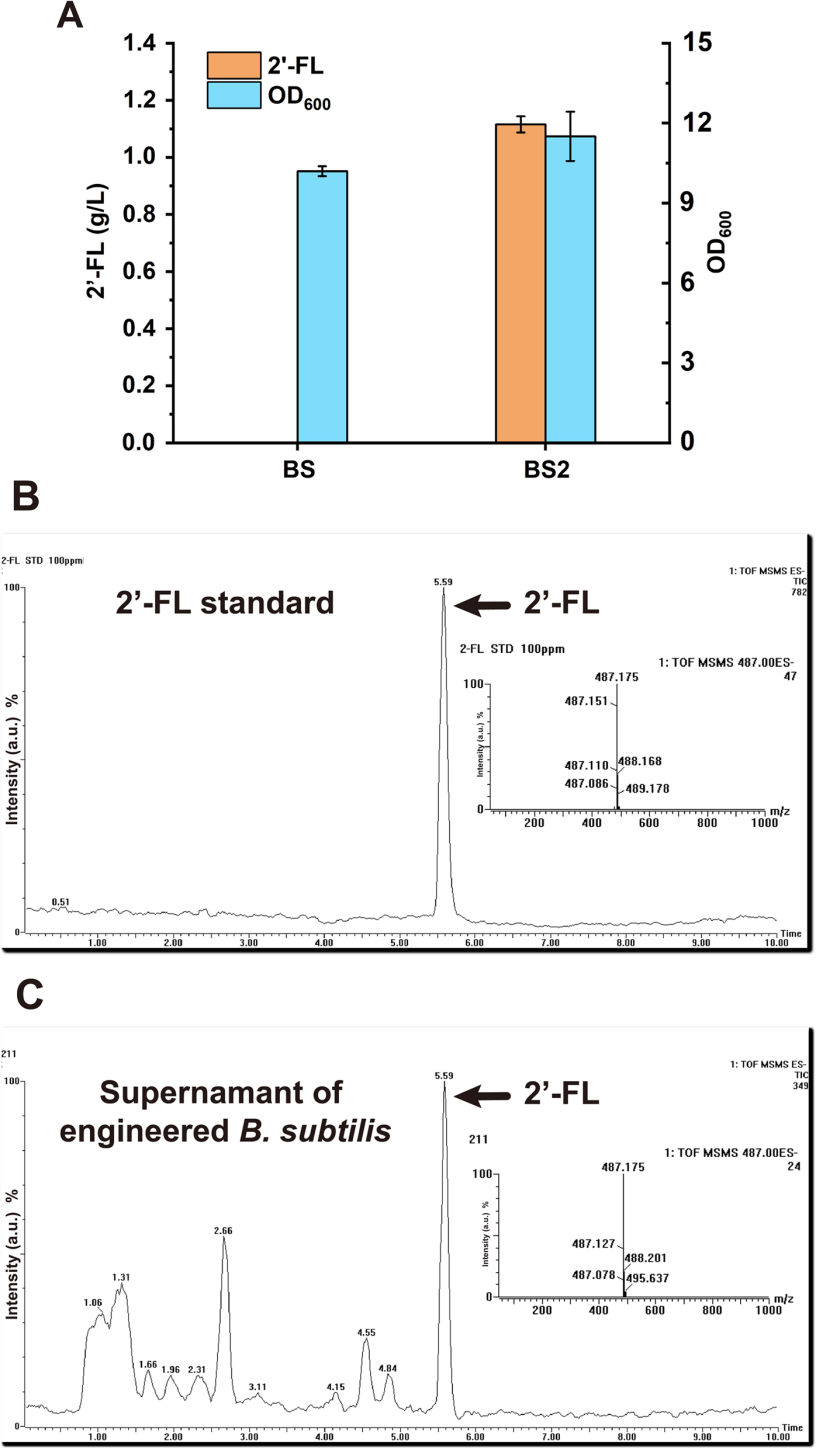
Production of 2′-FL in the strain BS2. **A** 2′-FL titer and OD_600_ of 20 g/L xylose-induced strain BS2. (**B** and **C**) 2′-FL standard and BS2 strain shake flask fermentation supernatant were analyzed by HPLC and LC/MS, respectively. Cells were cultivated for 36 h at 37 °C and 220 rpm. All data were the average of three independent studies with standard deviations

The P_43_ promoter is a strong constitutive promoter used to express various heterologous genes in *B. subtilis* [28, 29]. The above results indicated that the *manC* gene was not expressed, and eventually 2′-FL cannot be synthesized normally. It was speculated that the expression strength of the P_43_ promoter was not appropriate to ManC. Therefore, the inducible promoter P_*xylA*_ was used based on earlier reports to optimize the expression level of *manC* [30]. The accumulation of 1.12 g/L 2′-FL in the shake flask fermentation supernatant of strain BS2 (P_*xylA*_-*manC*) was achieved, whereas no 2′-FL was detected in the fermentation supernatant of the control strain BS (Fig. 3A). The LC/MS identification results are shown in Fig. 3B and C. Results showed that the inducible promoter P_*xylA*_ enabled the transcription and translation of *manC*, and 2′-FL was eventually synthesized in *B. subtilis*.

### Improving lactose utilization by knocking out *β*-galactosidase

**Fig. 4:**
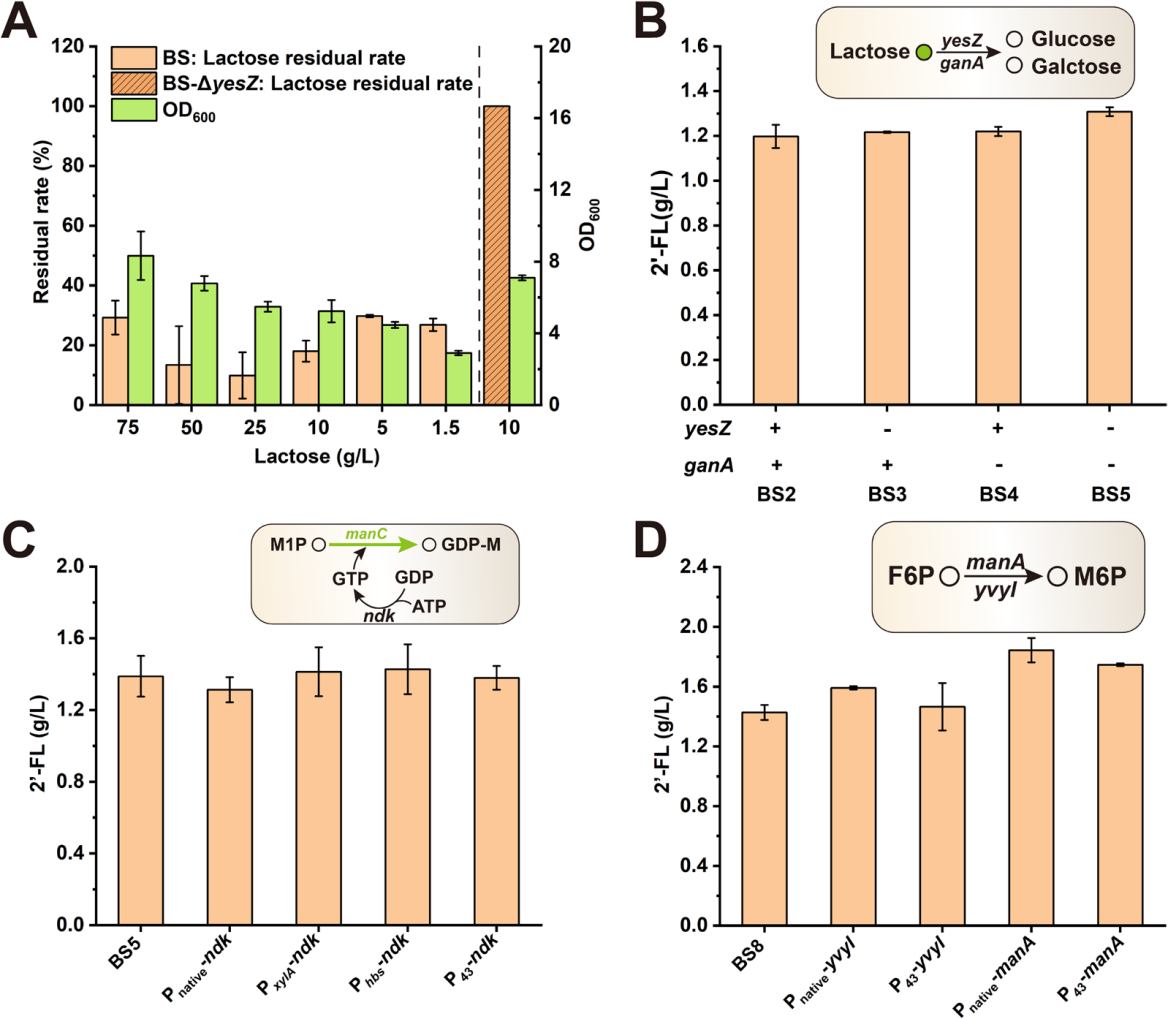
Increasing the supply of lactose, M6P and GTP. **A** The lactose utilization ability of wild-type *B. subtilis* and *yesZ* gene-deleted strain in 48-well plates (48 h). Symbol “ + ” means that this gene is present in the genome, and “ − ” indicates that this gene was deleted. **B** 2′-FL synthesis efficiency of strains with blocking lactose metabolism pathways was verified by fermentation. **C** Fermentation results of enhanced GTP supply experiments. **D** Fermentation results of enhanced precursor M6P supply experiments. All data were the average of three independent studies with standard deviations

As an important precursor for 2′-FL de novo synthesis, lactose is extremely important for the overproduction of 2′-FL. The lactose transportation in *B. subtilis* is dependent on the phosphotransferase system [31]. The *β*-galactosidase encoded by *yesZ* and *ganA* was able to hydrolyze lactose in *B. subtilis* [32, 33]. To test the lactose utilization ability of *B. subtilis, B. subtilis* was cultured in LB medium containing different concentrations of lactose (1.5, 5, 10, 25, 50, and 75 g/L). Figure 4A shows that *B. subtilis* consumed 70–90% of the lactose added in the medium. Among them, the experimental group with an initial lactose concentration of 75 g/L had the maximum residual lactose (~ 21.93 g/L) but with the consumption of > 50 g/L lactose. The final OD_600_ of *B. subtilis* gradually increased with the increase of lactose concentration, indicating that excess lactose was catabolized for cell growth (Fig. 4A). In addition, the effect of blocking the endogenous lactose metabolic pathway was tested by knocking out the *yesZ* gene, and results showed that *yesZ* deletion resulted in the cessation of lactose consumption in *B. subtilis* (Fig. 4A). According to the chemical reaction equation of 2′-FL synthesis, the stoichiometric ratio of lactose to 2′-FL was 1:1 (Fig. 1). Therefore, results indicated that the lactose transportation system of *B. subtilis* sufficed the needs of 2′-FL synthesis.

Although the lactose transport capacity was demonstrated, endogenous lactose metabolic pathways reduced the effective supply of lactose in *B. subtilis* (Fig. 1). *β*-Galactosidase could catalyze lactose hydrolysis; two *β*-galactosidase genes (*yesZ* and *ganA*) were found in *B. subtilis* [34–36]. Next, the effect of blocking the lactose catabolic pathway by knocking out different genes on 2′-FL accumulation was investigated. The results of shake flask fermentation (Fig. 4B) showed that knockout of the *yesZ* or *ganA* gene alone and in combination increased the 2′-FL titer to 1.22, 1.22, and 1.31 g/L, respectively. An earlier study suggested that the endogenous lactose transport system in *B. subtilis* was insufficient for the availability of 2′-FL production [20]. Results confirmed that the endogenous lactose transport system of *B. subtilis* sufficed the 2′-FL production with sufficient lactose, but a large amount of lactose was used for cell growth. Therefore, two *β*-galactosidase genes needed to be knocked out to improve lactose availability in *B. subtilis*.

### Enhancing 2′-FL production by engineering GTP and M6P supply

GTP and nicotinamide adenine dinucleotide phosphate were required as GDP donors and energy sources in the GDP-_*L*_-fucose de novo synthesis pathway (Fig. 1). 2′-FL production could be enhanced by increasing the GTP supply [20, 37, 38]. To increase GTP availability for optimal 2′-FL production, the *B. subtilis ndk* gene encoding a nucleoside-diphosphate kinase that converted GMP into GTP was respectively overexpressed by the original promoter (P_native_-*ndk*), inducible promoter (P_*xylA*_-*ndk*), and constitutive promoters (P_*hbs*_-*ndk* and P_43_-*ndk*). Shake flask fermentation results showed that the 2′-FL titer of strains BS7 (P_*xylA*_-*ndk*) and BS8 (P_*hbs*_-*ndk*) increased to 1.41 and 1.43 g/L, respectively, whereas the titers of the remaining strains decreased (Fig. 4C). Therefore, strain BS8 was selected for subsequent research.

The direct precursor of the de novo synthesis of GDP-_*L*_-fucose was M6P, which was catalyzed by the M6P isomerase encoded by *manA* and *yvyI* (putative) genes [39]. *manA* expression was regulated by mannose and a transcription activator (*manR*), and the endogenous promoter of *manA* was subjected to carbon catabolite repression [40]. To increase the supply of the precursor M6P, the original *manA* and *yvyI* were retained in the genome of strain BS8, and *manA* and *yvyI* expression cassettes harboring the original and strong promoters were introduced (P_native_-*yvyI*, P_43_-*yvyI*, P_native_-*manA* and P_43_-*manA*). Shake flask cultivation was performed for these strains with BS8 as a control. In Fig. 4D, the titers of the four strains (BS10–BS13) integrating the *manA* or *yvyI* gene increased to 1.59, 1.47, 1.84, and 1.75 g/L, respectively. Among these, with the P_native_-*manA* expression cassette, the titer increased most significantly up to 1.84 g/L. All gene insertions exhibited a positive effect on 2′-FL accumulation. Results indicated that the precursor M6P supply played an important role in 2′-FL production.

### Noninducible 2′-FL production and fermentation optimization

**Fig. 5:**
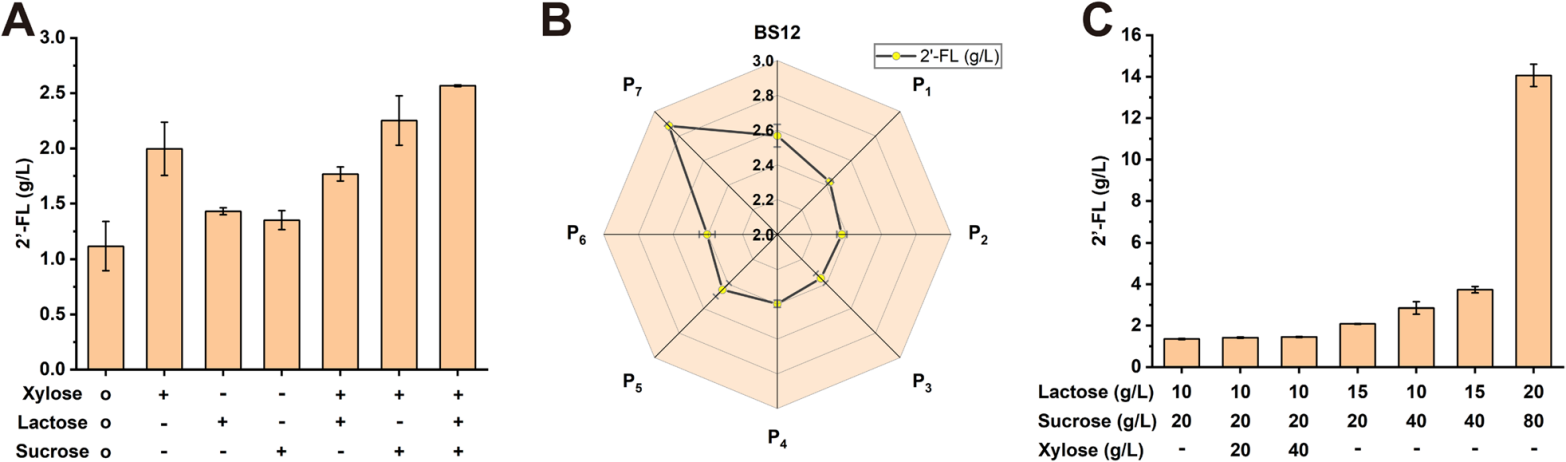
Fermentation optimization and optimization of the expression level of the *manC* gene. **A** and **B** Synthesis efficiency of 2′-FL was verified when the inducer xylose or carbon source (sucrose or lactose) was supplemented during 24-h fermentation using the BS12 strain. Symbol “o” indicates initial fermentation conditions (xylose 20 g/L, lactose 10 g/L, and sucrose 20 g/L). Symbol “ + ” indicates that 20 g/L xylose, lactose 10 g/L, and sucrose 20 g/L were supplemented when fermentation reached 24-h, and “–” means none of the three sugars were added. **C** Seven different constitutive promoters (P1–P7) were used to express the *manC* gene. **D** Optimization of fermentation conditions for strain BS20. All carbon sources were added at the beginning of fermentation. Symbol “ − ” means no added xylose. All data were the average of three independent studies with standard deviations

As mentioned in the preceding sections, a 2′-FL-producing strain with an improved titer was obtained. The titer and OD_600_ curves during the fermentation process (Additional file 1: Fig. S2) showed that 2′-FL accumulated slowly in the later stages of fermentation. Considering that this engineered strain required xylose as an inducer for 2′-FL production, there could be two possible reasons for the reduction in productivity volume: (1) *B. subtilis* could use xylose as a carbon source for growth and metabolite synthesis [41]. Therefore, xylose depletion as an inducer might have led to the inhibition of 2′-FL synthesis. (2) With the prolongation of the fermentation time, the residual carbon source was gradually consumed, and the lack of carbon source resulted in a decrease in production volume. Therefore, carbon source and inducer supplemental fermentation were performed. Results showed that adding the inducer xylose alone considerably increased the titer by 78.59% (2.00 g/L), and supplementing the lactose or sucrose alone increased the 2′-FL titer by 27.91% (1.43 g/L) and 20.77% (1.35 g/L), respectively (Fig. 5A). Results demonstrated that adding the inducer dose was the most effective in increasing the titer. In Fig. 5A, the 2′-FL titer increased by 129.72% with the addition of lactose and sucrose by increasing the inducer dose, and 2.57 g/L 2′-FL was obtained in the shake flask.

Although xylose supplementation significantly increased the 2′-FL titer, the high cost was unsuitable for an industrial-level production economy. To avoid using xylose, seven constitutive promoters (P1–P7) from *B. subtilis* with gradually increasing expression levels were used to replace inducible promoter P_*xylA*_. The fermentation results shown in Fig. 5B indicated that the titers of the strains harboring the P1 to P6 promoters decreased by 5.47%, 7.63%, 8.33%, 6.63%, 4.67%, and 6.36%, respectively. The strain BS20 harboring the strongest promoter had the highest 2′-FL titer, reaching 2.88 g/L (Fig. 5B). The highest 2′-FL titer was obtained by optimizing the addition of sucrose and lactose. In Fig. 5C, 80g/L sucrose and 20 g/L lactose were added at the beginning of the fermentation process, and the 2′-FL titer reached 14.06 g/L in 72 h (Fig. 5C). Meanwhile, considering that xylose could be consumed by *B. subtilis* as a carbon source, the effect of xylose on the 2′-FL titer was tested. Results showed that adding xylose did not affect 2′-FL accumulation (Fig. 5C).

### Enhancing 2′-FL production by engineering the transcriptional regulation of M6P isomerase

**Fig. 6:**
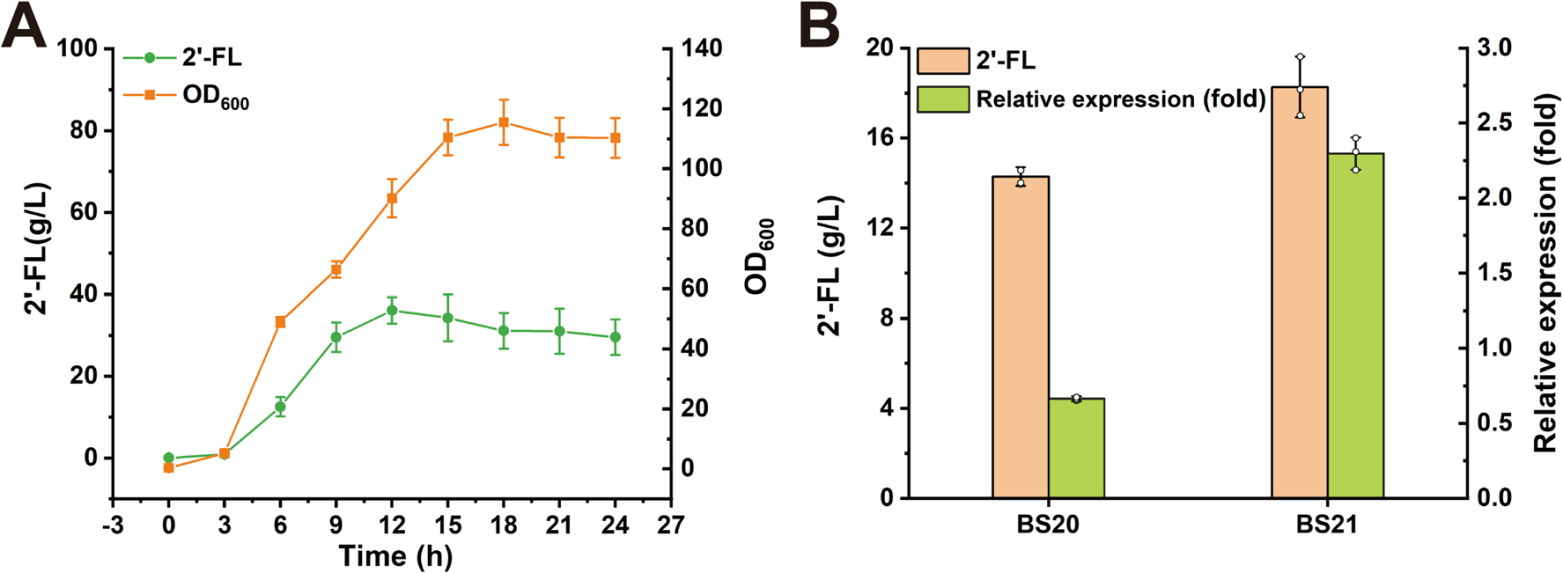
Engineering *manA* transcriptional regulation system to promote continuous synthesis of 2′-FL. **A** Fed-batch fermentation of strain BS20 in a 3-L bioreactor. 2′-FL synthesis stopped after 12 h fermentation. **B** The original promoter of *manA* was replaced to promote continuous 2′-FL production. qRT-PCR results showed that the expression level of the *manA* gene controlled by the constitutive promoter at 24 h was 2.297 times higher than that at 12 h. All data were the average of three independent studies with standard deviations

To further validate the 2′-FL production capacity of the engineered strain BS20, fermentation was performed in a 3-L bioreactor (Fig. 6A). Surprisingly, with the extension of fermentation time, the 2′-FL titer stopped increasing at 12 h, whereas the OD_600_ continued to increase, and a final 2′-FL titer of 36.09 g/L was obtained. All the exogenous genes in the 2′-FL de novo synthesis pathway was expressed with constitutive promoters, so there is no transcriptional repression for the expression of these genes. It was reported that, the endogenous *manA* gene (encoding the M6P isomerase) belongs to the mannose operon, and there is a phosphorylated transcription activator *manR* upstream of the mannose operon [40]. Therefore, we speculate that the stop of 2′-FL synthesis after 12 h may be due to the transcriptional inhibition of the endogenous *manA* gene.

Based on the above analysis, the original promoter (P_native_) of the *manA* gene of the BS20 strain was replaced with the constitutive promoter P7, generating the strain BS21. The cells of BS20 and BS21 cultured in shake flask for 12 and 24 h were collected, and the relative transcription level of the *manA* gene was analyzed by qRT-PCR (Fig. 6B). The results showed that the relative expression of *manA* at 24 h in the strain BS20 decreased to 0.664 times that at 12 h, whereas the relative expression of *manA* at 24 h in the strain BS21 increased to 2.297 times that at 12 h. Accordingly, the strain BS21 produced the highest 2′-FL titer of 18.27 g/L, which was 1.28-fold higher than that of the strain BS20 (14.29 g/L). This result suggested that the transcriptional regulation of *manA* hinders the continuous synthesis of 2′-FL in the later stage of fermentation, and the expression of *manA* gene with the constitutive promoter can solve this issue.

**Fig. 7:**
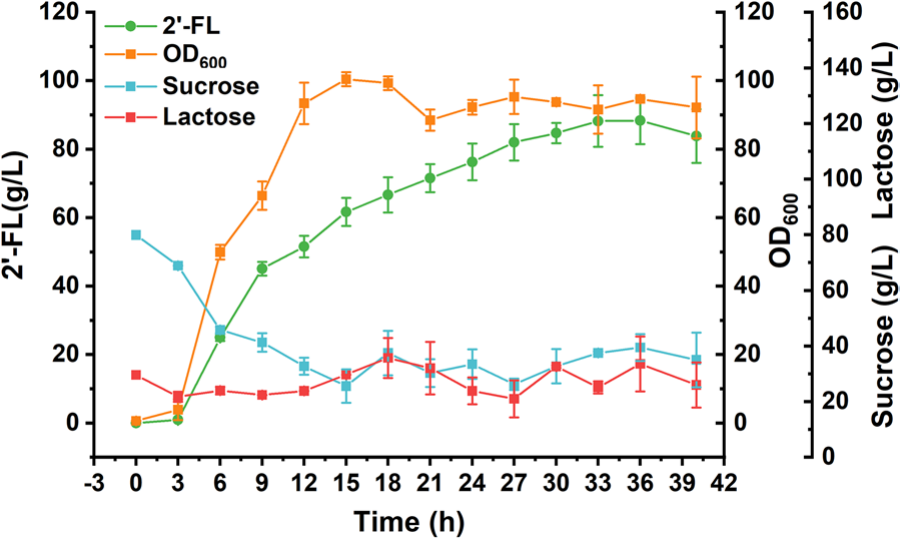
2′-FL production in a 3-L bioreactor. Fed-batch culture of the strain BS21 in a 3-L bioreactor. Sucrose and lactose were maintained at 10–30 g/L and 10–20 g/L, respectively. All data were the average of three independent studies with standard deviations

The production of 2′-FL by the finally engineered strain BS21 was performed in a 3-L bioreactor. The pH was kept at 6.0–6.5 by adding NH_4_OH; sucrose and lactose concentrations were maintained at 10–30 g/L and 10–20 g/L, respectively. Figure 7 shows that the strain BS21 could produce 88.3 g/L 2′-FL with a yield of 0.61 g/g lactose, representing the highest 2′-FL titer reported so far.

## Conclusions

This study constructed the 2′-FL de novo synthesis pathway in *B. subtilis* and further improved the 2′-FL titer by increasing the supply of M6P, lactose and cofactor GTP. In addition, the native promoter of *manA* gene was replaced with the constitutive promoter P7, leading to the further increase of 2′-FL titer. In a 3-L bioreactor, the 2′-FL titer of the finally engineered strain BS21 reached 88.3 g/L with a yield up to 0.61 mol/mol lactose. In the future, the dynamic regulation of central carbon metabolism with genetic circuits can be performed to further improve the synthesis efficiency of 2′-FL.

## Supplementary Information

Additional file 1: **Table S1.** Plasmids used in this study. **Table S2.** Primers and promoters used in this study. **Figure S1. **The SDS-PAGE analysis of cell extracts from single-gene expression strain. **Figure S2. **Production of 2′-FL in the shake flakes by the engineered strain BS2.

## Data Availability

The datasets used and/or analyzed during the current study are available from the corresponding authors on reasonable request.

## References

[1] Blanton LV, Charbonneau MR, Salih T, Barratt MJ, Venkatesh S, Ilkaveya O, Subramanian S, Manary MJ, Trehan I, Jorgensen JM (2016). Gut bacteria that prevent growth impairments transmitted by microbiota from malnourished children. Science.

[2] Shamash M, Maurice CF (2022). Phages in the infant gut: a framework for virome development during early life. ISME J.

[3] Sudheer S, Gangwar P, Usmani Z, Sharma M, Sharma VK, Sana SS, Almeida F, Dubey NK, Singh DP, Dilbaghi N (2022). Shaping the gut microbiota by bioactive phytochemicals: an emerging approach for the prevention and treatment of human diseases. Biochimie.

[4] Dogra SK, Martin FP, Donnicola D, Julita M, Berger B, Sprenger N (2021). Human milk oligosaccharide-stimulated bifidobacterium species contribute to prevent later respiratory tract infections. Microorganisms.

[5] Vandenplas Y, Berger B, Carnielli V, Ksiazyk J, Lagström H, Sanchez Luna M, Migacheva N, Mosselmans J-M, Picaud J-C, Possner M (2018). Human milk oligosaccharides: 2′-Fucosyllactose (2′-FL) and Lacto-*N*-neotetraose (LNnT) in infant formula. Nutrients.

[6] Zhang W, Liu Z, Gong M, Li N, Lv X, Dong X, Liu Y, Li J, Du G, Liu L (2021). Metabolic engineering of *Escherichia coli* for the production of Lacto-*N*-neotetraose (LNnT). Syst Microbiol and Biomanuf.

[7] Ballard O, Morrow AL (2013). Human milk composition: nutrients and bioactive factors. Pediatr Clin North Am.

[8] Donovan SM, Comstock SS (2016). Human milk oligosaccharides influence neonatal mucosal and systemic immunity. Ann Nutr Metab.

[9] Koromyslova A, Tripathi S, Morozov V, Schroten H, Hansman GS (2017). Human norovirus inhibition by a human milk oligosaccharide. Virology.

[10] Mohammad NS, Nazli R, Zafar H, Fatima S (2022). Effects of lipid based multiple micronutrients supplement on the birth outcome of underweight pre-eclamptic women: a randomized clinical trial. Pak J Med Sci.

[11] Wicinski M, Sawicka E, Gebalski J, Kubiak K, Malinowski B (2020). Human milk oligosaccharides: Health benefits, potential applications in infant formulas, and pharmacology. Nutrients.

[12] Ayechu-Muruzabal V, van Stigt AH, Mank M, Willemsen LEM, Stahl B, Garssen J, Van't Land B (2018). Diversity of Human milk oligosaccharides and effects on early life immune development. Front Pediatr.

[13] Petschacher B, Nidetzky B (2016). Biotechnological production of fucosylated human milk oligosaccharides: prokaryotic fucosyltransferases and their use in biocatalytic cascades or whole cell conversion systems. J Biotechnol.

[14] Zhou W, Jiang H, Wang L, Liang X, Mao X (2021). Biotechnological production of 2′-Fucosyllactose: a prevalent fucosylated Human milk oligosaccharide. ACS Synth Biol.

[15] Coyne MJ, Reinap B, Lee MM, Comstock LE (2005). Human symbionts use a host-like pathway for surface fucosylation. Science.

[16] Parschat K, Schreiber S, Wartenberg D, Engels B, Jennewein S (2020). High-titer *de novo* biosynthesis of the predominant Human milk oligosaccharide 2′-fucosyllactose from sucrose in *Escherichia coli*. ACS Synth Biol.

[17] Lee JW, Kwak S, Liu JJ, Yu S, Yun EJ, Kim DH, Liu C, Kim KH, Jin YS (2020). Enhanced 2′-Fucosyllactose production by engineered *Saccharomyces cerevisiae* using xylose as a co-substrate. Metab Eng.

[18] Jin-Ho S, Young-Wook C, Hae-Yong J (2020). *Corynebacterium glutamicum* for use in producing 2′-fucosyllactose.

[19] Zhang Q, Wu Y, Gong M, Zhang H, Liu Y, Lv X, Li J, Du G, Liu L (2021). Production of proteins and commodity chemicals using engineered *Bacillus subtilis* platform strain. Essays Biochem.

[20] Deng JY, Gu LY, Chen TC, Huang H, Yin XQ, Lv XQ, Liu YF, Li N, Liu ZM, Li JH (2019). Engineering the substrate transport and cofactor regeneration systems for enhancing 2′-fucosyllactose synthesis in *Bacillus subtilis*. ACS Synth Biol.

[21] Wu Y, Liu Y, Lv X, Li J, Du G, Liu L (2020). CAMERS-B: CRISPR/Cpf1 assisted multiple-genes editing and regulation system for *Bacillus subtilis*. Biotechnol Bioeng.

[22] Yu S, Liu JJ, Yun EJ, Kwak S, Kim KH, Jin YS (2018). Production of a human milk oligosaccharide 2′-fucosyllactose by metabolically engineered *Saccharomyces cerevisiae*. Microb Cell Fact.

[23] Wu Y, Chen T, Liu Y, Lv X, Li J, Du G, Ledesma-Amaro R, Liu L (2018). CRISPRi allows optimal temporal control of N-acetylglucosamine bioproduction by a dynamic coordination of glucose and xylose metabolism in *Bacillus subtilis*. Metab Eng.

[24] Lu Z, Yang S, Yuan X, Shi Y, Ouyang L, Jiang S, Yi L, Zhang G (2019). CRISPR-assisted multi-dimensional regulation for fine-tuning gene expression in *Bacillus subtilis*. Nucleic Acids Res.

[25] Salli K, Soderling E, Hirvonen J, Gursoy UK, Ouwehand AC (2020). Influence of 2′-fucosyllactose and galacto-oligosaccharides on the growth and adhesion of *Streptococcus mutans*. Br J Nutr.

[26] Weichert S, Jennewein S, Hüfner E, Weiss C, Borkowski J, Putze J, Schroten H (2013). Bioengineered 2′-fucosyllactose and 3-fucosyllactose inhibit the adhesion of *Pseudomonas aeruginosa* and enteric pathogens to human intestinal and respiratory cell lines. Nutr Res.

[27] Bode L, Contractor N, Barile D, Pohl N, Prudden AR, Boons GJ, Jin YS, Jennewein S (2016). Overcoming the limited availability of human milk oligosaccharides: challenges and opportunities for research and application. Nutr Rev.

[28] Wang PZ, Doi RH (1984). Overlapping promoters transcribed by *Bacillus subtilis* sigma 55 and sigma 37 RNA polymerase holoenzymes during growth and stationary phases. J Biol Chem.

[29] Wang J, Wang W, Wang H, Yuan F, Xu Z, Yang K, Li Z, Chen Y, Fan K (2019). Improvement of stress tolerance and riboflavin production of *Bacillus subtilis* by introduction of heat shock proteins from thermophilic bacillus strains. Appl Microbiol Biotechnol.

[30] Westbrook AW, Ren X, Oh J, Moo-Young M, Chou CP (2018). Metabolic engineering to enhance heterologous production of hyaluronic acid in *Bacillus subtilis*. Metab Eng.

[31] Reizer J, Bachem S, Reizer A, Arnaud M, Saier MH, Stülke J (1999). Novel phosphotransferase system genes revealed by genome analysis - the complete complement of PTS proteins encoded within the genome of *Bacillus subtilis*. Microbiology (Reading).

[32] Watzlawick H, Morabbi Heravi K, Altenbuchner J (2016). Role of the *ganSPQAB* operon in degradation of galactan by *Bacillus subtilis*. J Bacteriol.

[33] Carneiro L, Yu L, Dupree P, Ward RJ (2018). Characterization of a *β*-galactosidase from *Bacillus subtilis* with transgalactosylation activity. Int J Biol Macromol.

[34] Borriss R, Danchin A, Harwood CR, Médigue C, Rocha EPC, Sekowska A, Vallenet D (2018). *Bacillus subtilis*, the model Gram-positive bacterium: 20 years of annotation refinement. Microb Biotechnol.

[35] Shipkowski S, Brenchley JE (2006). Bioinformatic, genetic, and biochemical evidence that some glycoside hydrolase family 42 *β*-galactosidases are arabinogalactan type I oligomer hydrolases. Appl Environ Microbiol.

[36] Saqib S, Akram A, Halim SA, Tassaduq R (2017). Sources of *β*-galactosidase and its applications in food industry. Biotech.

[37] Ni Z, Wu J, Li Z, Yuan L, Wang Y, Chen X, Yao J (2021). Enhanced bioproduction of fucosylated oligosaccharide 3-fucosyllactose in engineered *Escherichia coli* with an improved *de novo* pathway. Biosci Biotechnol Biochem.

[38] Choi YH, Park BS, Seo JH, Kim BG (2019). Biosynthesis of the human milk oligosaccharide 3-fucosyllactose in metabolically engineered *Escherichia coli via* the salvage pathway through increasing GTP synthesis and *β*-galactosidase modification. Biotechnol Bioeng.

[39] Morabbi Heravi K, Manzoor I, Watzlawick H, de Jong A, Kuipers OP, Altenbuchner J (2019). Phosphosugar stress in *Bacillus subtilis*: Intracellular accumulation of mannose 6-phosphate derepressed the *glcR*-*phoC* operon from repression by GlcR. J Bacteriol.

[40] Sun T, Altenbuchner J (2010). Characterization of a mannose utilization system in *Bacillus subtilis*. J Bacteriol.

[41] Hu F, Liu Y, Lin J, Wang W, Li S (2020). Efficient production of surfactin from xylose-rich corncob hydrolysate using genetically modified *Bacillus subtilis* 168. Appl Microbiol Biotechnol.

