# Supplementary material for: Engineered Bacillus subtilis for the de novo production of 2′-fucosyllactose
Source: Microb Cell Fact. 2022 Jun 2;21:110. doi: 10.1186/s12934-022-01838-w (PMC9164505; doi:10.1186/s12934-022-01838-w)
Supplement: Supplementary file 1 — Additional file 1: Table S1. Plasmids used in this study. Table S2. Primers and promoters used in this study. Figure S1. The SDS-PAGE analysis of cell extracts from single-gene expression strain. Figure S2. Production of 2′-FL in the shake flakes by the engineered strain BS2. [file 12934_2022_1838_MOESM1_ESM.docx]

# Supporting materials for

# Engineered *Bacillus subtilis* for the *de novo* production of 2′-fucosyllactose

Quanwei Zhang^1,2^, Zhenmin Liu^3^, Hongzhi Xia^4^, Ziyang Huang^5^, Yonglian Zhu^1,2^, Linfeng Xu^1,2^, Yanfeng Liu^1,2^, Jianghua Li^1,2^, Guocheng Du^1,2^, Xueqin Lv^1,2,3^, Long Liu^1,2*^

^1^ Key Laboratory of Carbohydrate Chemistry and Biotechnology, Ministry of Education, Jiangnan University, Wuxi 214122, China.

^2^ Science Center for Future Foods, Jiangnan University, Wuxi 214122, China.

^3^ State Key Laboratory of Dairy Biotechnology, Shanghai Engineering Research Center of Dairy Biotechnology, Dairy Research Institute, Bright Dairy & Food Co., Ltd, Shanghai, 200436, China.

^4^ Nantong Licheng Biological Engineering Co., Ltd., Shanghai 200000, China.

^5^ Yixing Institute of Food Biotechnology Co., Ltd., Yixing, 214200, China.

^*^Corresponding author: Long Liu, Tel.: +86-510-85918312, Fax: +86-510-85918309, E-mail: longliu@jiangnan.edu.cn

Table S1 Plasmids used in this study

| Plasmid | Characterization | Source |
| --- | --- | --- |
| pcrF19NM | CRISPR/Cpf1 gene editing plasmid | lab stock |
| pHT-XCR6 | CRISPR/Cpf1 gene editing plasmid | ^1^ |
| pP43NMK | P_43_ promoter gene expression plasmid | lab stock |
| pcrF19NM-P_43_-*manB* | P_43_-*manB* expression cassette and crRNA | this work |
| pcrF19NM-P_43_-*manC* | P_43_-*manC* expression cassette and crRNA | this work |
| pcrF19NM-P_43_-*gmd* | P_43_-*gmd* expression cassette and crRNA | this work |
| pcrF19NM-P_43_-*wcaG* | P_43_-*wcaG* expression cassette and crRNA | this work |
| pcrF19NM-P_43_-*futC* | P_43_-*futC* expression cassette and crRNA | this work |
| pP43NMK-P_43_-*manB* | P_43_-*manB* expression cassette | this work |
| pP43NMK-P_43_-*manC* | P_43_-*manC* expression cassette | this work |
| pP43NMK-P_43_-*gmd* | P_43_-*gmd* expression cassette | this work |
| pP43NMK-P_43_-*wcaG* | P_43_-*wcaG* expression cassette | this work |
| pcrF19NM-P*_xylA_*-*manC* | P*_xylA_*-*manC* expression cassette and crRNA | this work |
| pcrF19NM-Δ*yesZ* | homologous arms (Δ*yesZ*) and crRNA | this work |
| pcrF19NM-Δ*ganA* | homologous arms (Δ*ganA*) and crRNA | this work |
| pcrF19NM-P_native_-*ndk* | P_native_-*ndk* expression cassette and crRNA | this work |
| pcrF19NM-P*_xylA_*-*ndk* | P*_xylA_*-*ndk* expression cassette and crRNA | this work |
| pcrF19NM-P*_hbs_*-*ndk* | P*_hbs_*-*ndk* expression cassette and crRNA | this work |
| pcrF19NM-P_43_-*ndk* | P_43_-*ndk* expression cassette and crRNA | this work |
| pcrF19NM-P_native_-*yvyI* | P_native_-*yvyI* expression cassette and crRNA | this work |
| pP43NMK-P_43_-*yvyI* | P_43_-*yvyI* expression cassette | this work |
| pcrF19NM-P_native_-*manA* | P_native_-*manA* expression cassette and crRNA | this work |
| pcrF19NM-P_1_ | P_1_::P*_xylA_* and crRNA | this work |
| pcrF19NM-P_2_ | P_2_::P*_xylA_* and crRNA | this work |
| pcrF19NM-P_3_ | P_3_::P*_xylA_* and crRNA | this work |
| pcrF19NM-P_4_ | P_4_::P*_xylA_* and crRNA | this work |
| pcrF19NM-P_5_ | P_5_::P*_xylA_* and crRNA | this work |
| pcrF19NM-P_6_ | P_6_::P*_xylA_* and crRNA | this work |
| pcrF19NM-P_7_ | P_7_::P*_xylA_* and crRNA | this work |

Table S2 Primers and promoters used in this study

| Primer | Sequence | Template or Description |
| --- | --- | --- |
| mB-UF | AGTTAGCTTGGCCAGTGCCTTTTGGAGAAGCGACAAGACGC | *B. subtilis* genome |
| mB-UR | AGTTAGCTTGGCCAGTGCCTTTTGGAGAAGCGACAAGACGC | *B. subtilis* genome |
| mB-P43U | CTTACTTTTCAAACCTATCCCTTGCTGAGTCTGGCTTTCGG | pP43NMK |
| mB-P43R | AGCTTTAAAGCAAGTTAGCATGTGTACATTCCTCTCTTACCTATAATGGTACCG | pP43NMK |
| mB-U | GGTAAGAGAGGAATGTACACATGCTAACTTGCTTTAAAGCTTATGATATTCGC | *E. coli* BL31(DE3) genome |
| mB-R | GTCGACCTGCAGTTAGTGGTGATGGTGATGATGCTTGTTCA | *E. coli* BL31(DE3) genome |
| mB-DF | ATCACCATCACCACTAACTGCAGGTCGACGTCCCC | *B. subtilis* genome |
| mB-DR | GATCTGCCGTTCGTAACAGGTTGCTGATGATAATCGAGAGCTG | *B. subtilis* genome |
| pcrF19NM-mB-F | CAGCTCTCGATTATCATCAGCAACCTGTTACGAACGGCAGA | pcrF19NM |
| pcrF19NM-mB-R | TCGCTTCTCCAAAAGGCACTGGCCAAGCTAACTAAGT | pcrF19NM |
| mB-crRNA-F | AGATCTATATGCGGATACTTAATATTC | synthetic DNA |
| mB-crRNA-R | AATTGAATATTAAGTATCCGCATATAG | synthetic DNA |
| mC-UF | AGTTAGCTTGGCCAGTGCCTAGAAACTCCTGGGCTTTTATCCC | *B. subtilis* genome |
| mC-UR | GACTCAGCAAGCAGGCACCCTTGTTAGCTGT | *B. subtilis* genome |
| mC-P43U | ACAAGGGTGCCTGCTTGCTGAGTCTGGCTTTCGG | pP43NMK |
| mC-P43R | GGAATAAGAGGTGAGCTCATGTGTACATTCCTCTCTTACCTATAATGGTACCG | pP43NMK |
| mC-U | GGTAAGAGAGGAATGTACACATGAGCTCACCTCTTATTCCGGTTAT | *E. coli* BL31(DE3) genome |
| mC-R | CGACCTGCAGTCAGTGGTGATGGTGATGATGATCTTCAAATC | *E. coli* BL31(DE3) genome |
| mC-DF | ACCATCACCACTGACTGCAGGTCGACGTCCCC | *B. subtilis* genome |
| mC-DR | TGATCTGCCGTTCGTAACAGGTGTGTTGAACTATTGAACAGCGG | *B. subtilis* genome |
| pcrF19NM-mC-F | CAATAGTTCAACACACCTGTTACGAACGGCAGATCAGAA | pcrF19NM |
| pcrF19NM-mC-R | CAGGAGTTTCTAGGCACTGGCCAAGCTAACTAAGTTTGAA | pcrF19NM |
| mC-crRNA-F | AGATGGCACCCTTGTTCTTTAAAAAAG | synthetic DNA |
| mC-crRNA-R | AATTCTTTTTTAAAGAACAAGGGTGCC | synthetic DNA |
| gd-UF | CTTGGCCAGTGCCTCTCCTAGTGCCGTCATATGTTAAAGTTAGAGG | *B. subtilis* genome |
| gd-UR | CCAGACTCAGCAAGACATCCGCTCATAACAACAGCG | *B. subtilis* genome |
| gd-P43U | TGTTGTTATGAGCGGATGTCTTGCTGAGTCTGGCTTTCGG | pP43NMK |
| gd-P43R | ATGTAAAATATAAAGTGATAGCGGTACCATTATAGGAGGAGTTTACATGTCAAAAGTCGCTCTCA | pP43NMK |
| gd-U | CGGTGATGAGAGCGACTTTTGACATGTAAACTCCTCCTATAATG | *E. coli* BL31(DE3) genome |
| gd-R | CACTTGGGGAAACCCTAGTGGTGATGGTGATGATGTGACTCCAGCG | *E. coli* BL31(DE3) genome |
| gd-DF | ACCATCACCACTAGGGTTTCCCCAAGTGTTTAGTGACATGA | *B. subtilis* genome |
| gd-DR | GATCTGCCGTTCGTAACAGGAGACTTCCAGCCTAATGCTAC | *B. subtilis* genome |
| pcrF19NM-gd-F | ATAGCAGTAGCATTAGGCCCTGTTACGAACGGCAGATCAGAATTTTGTAATA | pcrF19NM |
| pcrF19NM-gd-R | ATGACGGCACTAGGAGAGGCACTGGCCAAGCTAACTAAGTT | pcrF19NM |
| gd-crRNA-F | AGATAATACATGAATTTGATGCTGGTG | synthetic DNA |
| gd-crRNA-R | AATTCACCAGCATCAAATTCATGTATT | synthetic DNA |
| wG-UF | AGTTAGCTTGGCCAGTGCCTTCAAAGCGGAGAAACAGCAGACAG | *B. subtilis* genome |
| wG-UR | ACTCAGCAAGGGCTCTCCGGTTTTCGGATTTC | *B. subtilis* genome |
| wG-P43U | ACCGGAGAGCCCTTGCTGAGTCTGGCTTTCGG | pP43NMK |
| wG-P43R | ACTCGTTGTTTACTCATGTGTACATTCCTCTCTTACCTATAATGGTACCG | pP43NMK |
| wG-U | GTACCATTATAGGTAAGAGAGGAATGTACACATGAGTAAACAACGAGTTTTTATTGCTGGTC | *E. coli* BL31(DE3) genome |
| wG-R | ACATTGTAAGTGCGTCAGTGGTGATGGTGATGATGCC | *E. coli* BL31(DE3) genome |
| wG-DF | CACCATCACCACTGACGCACTTACAATGTACTTGTGGGAT | *B. subtilis* genome |
| wG-DR | GATCTGCCGTTCGTAACAGGACGCGTTTTTTATATCTTGGGCCAT | *B. subtilis* genome |
| pcrF19NM-wG-F | CCTGTTACGAACGGCAGA | pcrF19NM |
| pcrF19NM-wG-R | CTCCGCTTTGAAGGCACTGGCCAAGCTAACTAAGTTTGA | pcrF19NM |
| wG-crRNA-F | AGATTTGACAATTAATAAAGGGCAAAG | synthetic DNA |
| wG-crRNA-R | AATTCTTTGCCCTTTATTAATTGTCAA | synthetic DNA |
| fC-UF | AGCTTGGCCAGTGCCTATACAGGTCCATCTTGGAAACTGTCCCGC | *B. subtilis* genome |
| fC-UR | CCAGACTCAGCAAGGGATCATTGGGAGCAGCAGGC | *B. subtilis* genome |
| fC-P43U | GCTCCCAATGATCCCTTGCTGAGTCTGGCTTTCGG | pP43NMK |
| fC-P43R | AACAACCTTAAATGCCATGTGTACATTTCACCTCCTTTGGTACCGCTATCACTTTATATTTTACAT | pP43NMK |
| fC-U | GGAGGTGAAATGTACACATGGCATTTAAGGTTGTTCAGATTTGC | *E. coli* BL31(DE3) genome |
| fC-R | AGCGCTAAAGTTAGTGGTGATGGTGATGATGTGCATTATATTTTTGTGATTTAACTTCAAAATGTGATTCAAT | *E. coli* BL31(DE3) genome |
| fC-DF | ACATCATCACCATCACCACTAACTTTAGCGCTCCTTTCATTTAAAAGGTG | *B. subtilis* genome |
| fC-DR | CCGTTCGTAACAGGCCAGCTGTCATCGCGAACAACTTTGACT | *B. subtilis* genome |
| pcrF19NM-fC-F | TCGCGATGACAGCTGGCCTGTTACGAACGGCAGATCAGAAT | pcrF19NM |
| pcrF19NM-fC-R | GACCTGTATAGGCACTGGCCAAGCTAACTAAGTTTGAAA | pcrF19NM |
| fC-crRNA-F | AGATCAATCGTACACATGAGATTCACG | synthetic DNA |
| fC-crRNA-R | AATTCGTGAATCTCATGTGTACGATTG | synthetic DNA |
| PxylA-mC-F | GGAAATGGGATCCATGAGCTCACCTCTTATTCCGGTTAT | pcrF19NM-P_43_-*manC* |
| PxylA-mC-R | CGACCTGCAGTCAGTGGTGATGGTGATGATGATCTTCAAATC | pcrF19NM-P_43_-*manC* |
| PxylA-F | ACCATCACCACTGACTGCAGGTCGACGTCCCC | synthetic DNA |
| PxylA-R | CGGAATAAGAGGTGAGCTCATGGATCCCATTTCCCCCTTTGATTTTTAGATATCA | synthetic DNA |
| ΔyesZ-UF | CGTATCTATACAACAACACATCTCAC | *B. subtilis* genome |
| ΔyesZ-UR | TTCGATACAACACATTTTCACCTTTCTTTGATGAT | *B. subtilis* genome |
| ΔyesZ-DF | TGAAAATGTGTTGTATCGAATCAGCTTTTTTCTATGG | *B. subtilis* genome |
| ΔyesZ-DR | GATTTCCAGTGCTGAAGGATAT | *B. subtilis* genome |
| ΔyesZ-crRNA-F | AGATTTAAAACTGAGCCTCACCGGGGA | synthetic DNA |
| ΔyesZ-crRNA-F | AATTTCCCCGGTGAGGCTCAGTTTTAA | synthetic DNA |
| ΔganA-UF | GCTCCGATTATCATTACTCAGTTTACGT | *B. subtilis* genome |
| ΔganA-UR | GCGGAGCATCAGCTTAGCCCTTCGTTCCGCCTG | *B. subtilis* genome |
| ΔganA-DF | CGAAGGGCTAAGCTGATGCTCCGCTCGATATG | *B. subtilis* genome |
| ΔganA-DR | GTATTCAGCAGCATAGCTGGACG | *B. subtilis* genome |
| ΔganA-crRNA-F | AGATTCATACGAATACGTTTTCTGTCG | synthetic DNA |
| ΔganA-crRNA-F | AATTCGACAGAAAACGTATTCGTATGA | synthetic DNA |
| P1mCPD-F | CTGAGAAAGGAGGTGATAAAAATGAGCTCACCTCTTATTCCGGTTAT | synthetic DNA |
| P1mCPD-R | GCGATTAAGTTGGGTCAGTGGTGATGGTGATGATGATCTTCAAATC | synthetic DNA |
| P1mCZT-F | ATCACCATCACCACTGACCCAACTTAATCGCCTTGCAGCAC | pcrF19NM-P_43_-*manC* |
| P1mCZT-R | GGAATAAGAGGTGAGCTCATTTTTATCACCTCCTTTCTCAGTTTTAATATTATTATCTACTACG | pcrF19NM-P_43_-*manC* |
| P2mCPD-F | ATGTGACTATAAAGGAGGTGATAAAAATGAGCTCACCTCTTATTCCGGTTAT | synthetic DNA |
| P2mCPD-R | GCGATTAAGTTGGGTCAGTGGTGATGGTGATGATGATCTTCAAATC | synthetic DNA |
| P2mCZT-F | ATCACCATCACCACTGACCCAACTTAATCGCCTTGCAGCAC | pcrF19NM-P_43_-*manC* |
| P2mCZT-R | AGAGGTGAGCTCATTTTTATCACCTCCTTTATAGTCACATTTATTTTTACGC | pcrF19NM-P_43_-*manC* |
| P3mCPD-F | CATAATGAAAGTAATTTAAAGGAGGTGATAAAAATGAGCTCACCTCTTATTCCGGTTAT | synthetic DNA |
| P3mCPD-R | GCGATTAAGTTGGGTCAGTGGTGATGGTGATGATGATCTTCAAATC | synthetic DNA |
| P3mCZT-F | ATCACCATCACCACTGACCCAACTTAATCGCCTTGCAGCAC | pcrF19NM-P_43_-*manC* |
| P3mCZT-R | AGAGGTGAGCTCATTTTTATCACCTCCTTTAAATTACTTTCATTATGAGTTAAATTTCC | pcrF19NM-P_43_-*manC* |
| P4mCPD-F | GAGATTAAAGGAGGTGATAAAAATGAGCTCACCTCTTATTCCGGTTAT | synthetic DNA |
| P4mCPD-R | GCGATTAAGTTGGGTCAGTGGTGATGGTGATGATGATCTTCAAATC | synthetic DNA |
| P4mCZT-F | ATCACCATCACCACTGACCCAACTTAATCGCCTTGCAGCAC | pcrF19NM-P_43_-*manC* |
| P4mCZT-R | CGGAATAAGAGGTGAGCTCATTTTTATCACCTCCTTTAATCTCTTCCCCCACTTC | pcrF19NM-P_43_-*manC* |
| P5mCPD-F | ATCGTTGAAAGGAGGTGATAAAAATGAGCTCACCTCTTATTCCGGTTAT | synthetic DNA |
| P5mCPD-R | GCGATTAAGTTGGGTCAGTGGTGATGGTGATGATGATCTTCAAATC | synthetic DNA |
| P5mCZT-F | ATCACCATCACCACTGACCCAACTTAATCGCCTTGCAGCAC | pcrF19NM-P_43_-*manC* |
| P5mCZT-R | ATCGTTGAAAGGAGGTGATAAAAATGAGCTCACCTCTTATTCCGGTTAT | pcrF19NM-P_43_-*manC* |
| P6mCPD-F | AGATACATATCCTTAATAAAGGAGGTGATAAAAATGAGCTCACCTCTTATTCCGGTTAT | synthetic DNA |
| P6mCPD-R | GCGATTAAGTTGGGTCAGTGGTGATGGTGATGATGATCTTCAAATC | synthetic DNA |
| P6mCZT-F | ATCACCATCACCACTGACCCAACTTAATCGCCTTGCAGCAC | pcrF19NM-P_43_-*manC* |
| P6mCZT-R | AGAGGTGAGCTCATTTTTATCACCTCCTTTATTAAGGATATGTATCTATTTCTCT | pcrF19NM-P_43_-*manC* |
| P7mCPD-F | ATCAGAAAGGAGGTGATAAAAATGAGCTCACCTCTTATTCCGGTTAT | synthetic DNA |
| P7mCPD-R | GCGATTAAGTTGGGTCAGTGGTGATGGTGATGATGATCTTCAAATC | synthetic DNA |
| P7mCZT-F | ATCACCATCACCACTGACCCAACTTAATCGCCTTGCAGCAC | pcrF19NM-P_43_-*manC* |
| P7mCZT-R | CGGAATAAGAGGTGAGCTCATTTTTATCACCTCCTTTCTGATAATATAACATATTCTCA | pcrF19NM-P_43_-*manC* |
| Promoter P1 | TCATAGACCTGAAAAGGTCTTTTTTTGTACTCTTAATAATAAAAAGAAGATGAAACTTGTTTAAGGATTGAACGTAGTAGATAATAATATTAAAACTGAGAAAGGAGGTGATAAAA |  |
| Promoter P2 | ATTATTCTTAACTTTTACGAAACTTTGATATAATAACAAACGTATATATTAGTAATTTACGGCTTATTTTCCTTGTGAGCGTAAAAATAAATGTGACTATAAAGGAGGTGATAAAA |  |
| Promoter P3 | AAACAATGAAACTTTTTTTTATAAAAAACGACTATTTTAGGATTTCATTCTTGTATTAAATAGAGTTGTATTTATTGGAAATTTAACTCATAATGAAAGTAATTTAAAGGAGGTGATAAAA |  |
| Promoter P4 | TTTTCTTGACGCCCTTTTGAGGGAGGAGTAAAATGAAATTGTCAATAAATCTTAATAAAGTGCTTACAATTGAAAGAAGTGGGGGAAGAGATTAAAGGAGGTGATAAAA |  |
| Promoter P5 | TTTTCGAATGATTAAATTTTTTGTTTTTTATAAAGGTTTTTTACTATTTTGTGAACAATCAAGGTAGAATCAAATTGCAAACAGTGGTAAAATATCGTTGAAAGGAGGTGATAAAA |  |
| Promoter P6 | AAACAAAATTCGACAAAGTTCACTGAATTTTCACAAAAGATTTATGTTTCAGCAGGAATTGTAAAGGGTAAAAGAGAAATAGATACATATCCTTAATAAAGGAGGTGATAAAA |  |
| Promoter P7 | AAAAAACGGCCTCTCGAAATAGAGGGTTGACACTCTTTTGAGAATATGTTATATTATCAGAAAGGAGGTGATAAAA |  |

## Figure legends

**Figure S1. The SDS-PAGE analysis of cell extracts from single-gene expression strain.** The white rectangular frame indicates the target protein in the supernatant after cell disruption.

**Figure S2. Production of 2′-FL in the shake flakes by the engineered strain BS2.**

Figure S1.


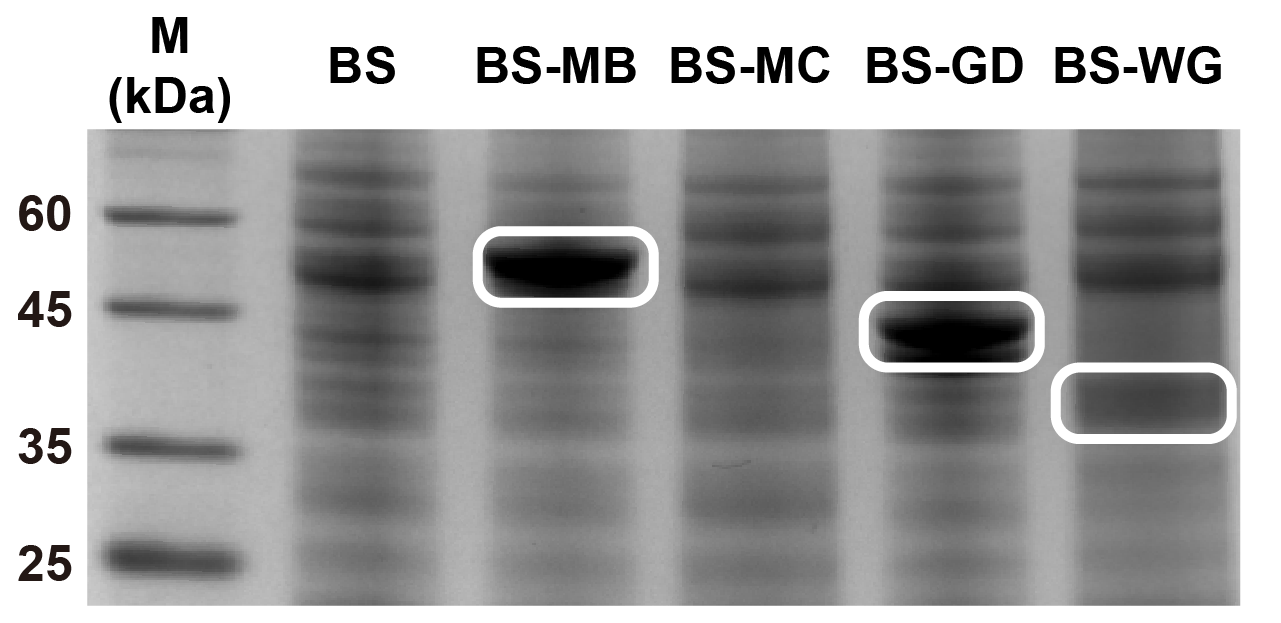


Figure S2.


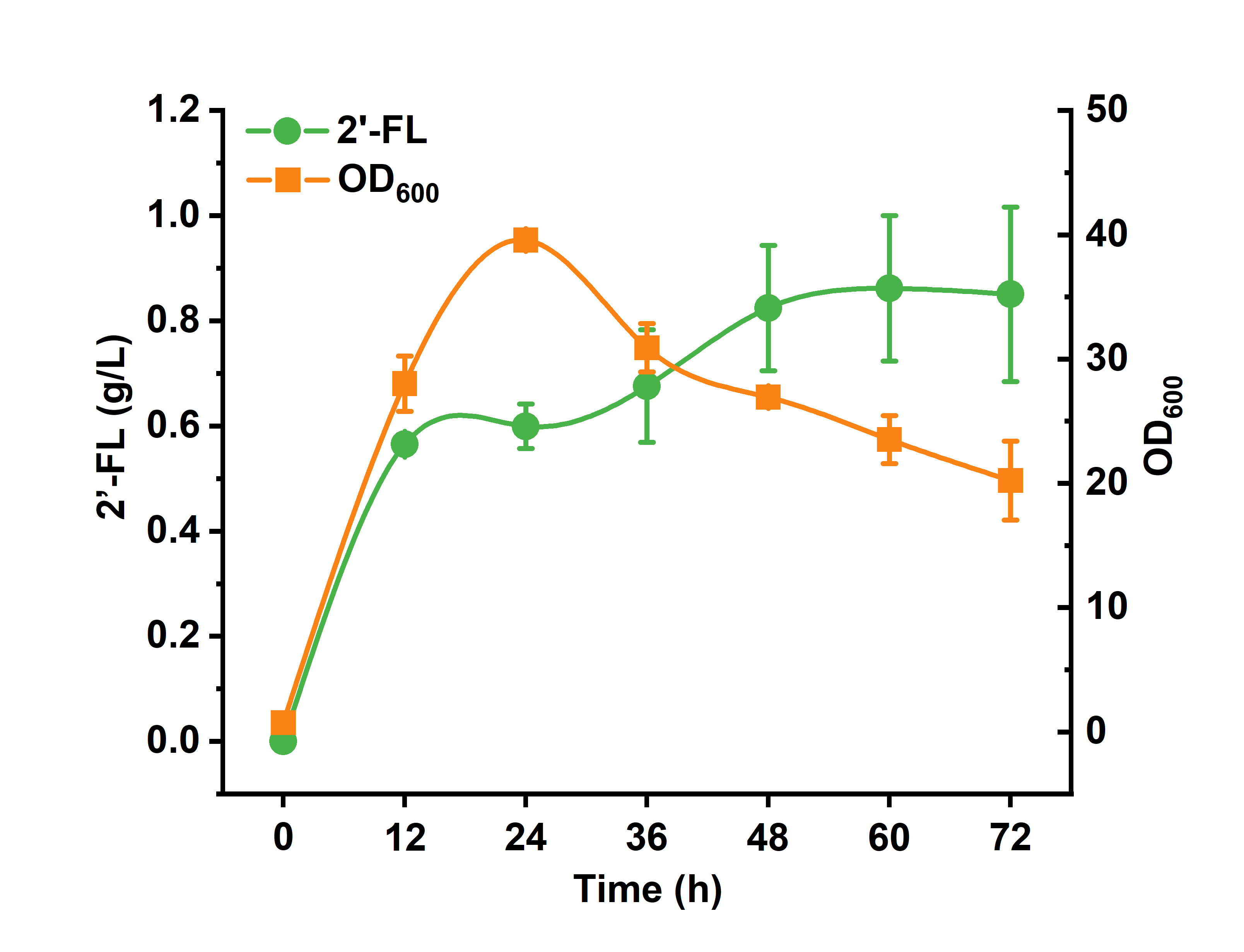


## References

(1) Wu, Y.; Liu, Y.; Lv, X.; Li, J.; Du, G.; Liu, L. CAMERS-B: CRISPR/Cpf1 assisted multiple-genes editing and regulation system for *Bacillus subtilis*. *Biotechnology and bioengineering* **2020**, *117* (6), 1817-1825. DOI: 10.1002/bit.27322.
